# Biodegradable Iron-Based Materials—What Was Done and What More Can Be Done?

**DOI:** 10.3390/ma14123381

**Published:** 2021-06-18

**Authors:** Gabriela Gąsior, Jonasz Szczepański, Aleksandra Radtke

**Affiliations:** Faculty of Chemistry, Nicolaus Copernicus University in Toruń, Gagarina 7, 87-100 Toruń, Poland; ggasior@doktorant.umk.pl (G.G.); jszcze@gmail.com (J.S.)

**Keywords:** iron, corrosion rate, biodegradable material, biocompatibility, stent

## Abstract

Iron, while attracting less attention than magnesium and zinc, is still one of the best candidates for biodegradable metal stents thanks its biocompatibility, great elastic moduli and high strength. Due to the low corrosion rate, and thus slow biodegradation, iron stents have still not been put into use. While these problems have still not been fully resolved, many studies have been published that propose different approaches to the issues. This brief overview report summarises the latest developments in the field of biodegradable iron-based stents and presents some techniques that can accelerate their biocorrosion rate. Basic data related to iron metabolism and its biocompatibility, the mechanism of the corrosion process, as well as a critical look at the rate of degradation of iron-based systems obtained by several different methods are included. All this illustrates as the title says, what was done within the topic of biodegradable iron-based materials and what more can be done.

## 1. Introduction

Coronary artery disease (CAD) is characterised by narrowing of the blood vessels that supply oxygenated blood to cardiac muscles: it is responsible for around 20% of all deaths in developed countries [1]. In 1977, for the first time, an angioplasty was performed. This procedure, using a balloon inserted into a narrowed blood vessel and then inflated, enabled the vessel to be restored and prolonged the life of the first 38-year-old patient by 37 years [2] Balloon angioplasty, however, was limited by unpredictable vessel dissection and recoil and by the high rate of restenosis [3]. Therefore, the next revolution in the treatment of cardiovascular medicine was the introduction of stents, which resulted in both better early results and lower rates of restenosis. At the same time, there were limitations due to stent thrombosis and neointimal hyperplasia resulting in vasoconstriction [4].

The first coronary stent made from stainless steel used in surgery was introduced in 1987 by Siegward [5]. Currently, the clinical uses of coronary stents are made from either 316 L stainless steel, Co–Cr or TiNi alloys [3]. There are two main types of metal stents: self-expanding stents, confined by a sheath that can be removed after delivery of the device, and balloon-expandable stents that are mounted on a balloon catheter that is inflated to deploy the device. Self-expanding stents have technical limitations and a tendency to induce greater neointimal hyperplasia. Therefore, balloon-expandable stents are used in most coronary stent procedures [2].

In 2007, Mani et al. formulated nine features that an ideal stent should have: (1) good expandability ratio; (2) ability to be crimped on the balloon catheter; (3) sufficient flexibility; (4) sufficient radial hoop strength and negligible recoil; (5) non-toxicity for tissues and all organisms; (6) high thromboresistivity; (7) absence of restenosis after implantation; (8) drug delivery capacity; and (9) adequate radiopacity/magnetic resonance imaging (MRI) compatibility [6].

In the meantime, the idea of biodegradable (or bioresorbable) stents arose. The biggest advantage of biodegradable stents (BDS) is that they disappear when they are no longer needed, which is about six months after the implantation. In this way, all late stent complications, like permanently diminished flow of covered side branches, bleeding problems associated with long term anticoagulation, permanent late fracture abnormal vasomotion and CT/MRI imaging artefacts are omitted [7,8,9,10,11]. At the same time, BDS provides mechanical support analogous to bare-metal stents. It is also a better solution for still-growing children because it helps avoids a second intervention to remove the implant [12]. This is why the list of features of an ideal stent should include a tenth feature: fully biodegradable.

Two types of materials are used to create bioresorbable stents or scaffolds (BRS): polymers and metals. Initially, more attention was paid to polymers, and already in 1998, so over 20 years ago, a scaffold composed of high-molecular-weight poly-l-lactic acid (PLLA) monofilaments) was implanted per Igaki-Tamai into a human coronary artery [13]. The first report, where a total of 25 scaffolds were successfully implanted into 19 lesions of 15 patients, were described and published in 2000 [13]. Long-term (>10 years) studies in 50 patients showed that, after three years, no traces of the stent scaffold in the blood vessel could be detected [14]. The results were great, but the device failed to progress as it required a larger guide catheter for implantation than a metal stent, it needed a heated contrast, and it had the lack of a drug coating [14]. However, the proposal to use PLLA in stents was not forgotten, and research is still ongoing. In 2016, the Absorb drug-eluting device, based on PLLA, was the first bioresorbable cardiovascular scaffold approved for use in the United States by the U.S. Food and Drug Administration [2]. Other biodegradable polymers and copolymers used for research include: poly(ε-caprolactone) (PCL), poly(l-lactide-co-ε-caprolactone) (PLCL), phosphoryl choline (ChoP), poly(desaminotyrosyl-tyrosine ethyl ester) carbonate (PTD-PC), poly(anhydride ester) (PAE), poly(d,l-lactide-co-glycolide) (PLGA), and poly(vinylidenefluoride)-hexafluoropropylene (PVDF-HFP) [6,13,15,16,17,18]. Despite very promising results, the polymers also have several disadvantages that limit their use. Compared to metals, polymers have lower values of Young’s modulus (0.2–7.0 GPa) than those of metals (54–200 GPa), and generally have poorer mechanical properties [19]. This makes the spacers in polymer stents thicker than in metal stents, which results in the impossibility of complete expansion as the balloon expands [2]. Therefore, more and more research is being done to create a biodegradable metal stent. This would allow the advantage of polymeric and metallic devices to be combined.

Metals degrade in the body through corrosion. Therefore, metals used in first-generation stents, such as stainless steel, nitinol or titanium, which have a high corrosion resistance factor, cannot be used as resorbable materials. From research conducted over the past 20 years, three main metals have emerged that could potentially form biodegradable cardiovascular implants: Fe, Mg and Zn [20,21,22]. This review summarises the achievements in developing fully biodegradable iron-based stents, but the most important features of the next two metals should be mentioned. Magnesium BDS are completely biocompatible and have good mechanical properties. However, magnesium has a high corrosion rate, which means it loses integrity when it is still needed. Moreover, it releases hydrogen during degradation that is harmful to cells. Despite their drawbacks, magnesium-based devices were the first to be approved for clinical trials and are now commercially available, for example, in Biotronik’s Magmaris [23]. Zinc is characterised by good biocompatibility and a corrosion factor adequate to the desired lifetime of the stent. However, its mechanical properties are too weak (Table 1). It is necessary to introduce modifications to improve the mechanical parameters and, at the same time, not affect the corrosion time [24].

Iron has a high strength, ductility, and formability (Table 1), allowing stents with thinner constructions and struts or fabrication of special shapes, like foils or foams [25]. Unfortunately, in comparison to Zn and Mg, iron has a corrosion rate so low that pure iron can hardly be called “biodegradable”. But due to its biocompatibility and excellent mechanical properties, it is worth thinking about modifications that could accelerate corrosion.

In the following sections of the article, readers will find the information about the iron properties, its biodegradability and its corrosion test, which can be carried out in immersion mode and during the electrochemical testing. Biological properties of iron-based materials, in terms of tissue biocompatibility, cellular biocompatibility, hemocompatibility, and clinical biocompatibility are presented and discussed also. A critical look at the rate of degradation of systems obtained by several different synthesis methods, including: spark plasma sintering, vacuum induction melting, vacuum arc melting, electroforming, powder metallurgy and template-based synthesis of porous materials, as well as by the addition of another phase to the iron, will allow the reader to select methods which are still worth optimizing because they give hope for their use in biomedical applications, and those that they do not provide any chance of obtaining iron-based material as an optimally biodegradable system.

## 2. Biodegradability of Pure Iron

In 2001, the first results of an in vivo study by Peuster et al. showed that pure iron degrades too slowly in the body to be considered a bioabsorbable material [31]. There was a need to modify the iron to increase its corrosion rate without losing its biocompatibility and good mechanical properties. Since then, several studies have been carried out to understand how the working environment of an implant affects the rate of corrosion and the products that result from this process.

In a physiological environment, the corrosion rate of Fe highly depends on the rate of the cathodic reaction and the amount of dissolved oxygen. An increase in the oxygen levels in the corrosive environment will also increase the iron corrosion rate. In contrast, a decrease in the amount of oxygen in contact with Fe causes a reduction in the corrosion rate [32]:

Fe → Fe^2+^ + 2e^−^ (anodic reaction) (1)

Electrochemical equations for iron corrosion work as follows [33]:

O_2_ + 2H_2_O + 4e^−^ → 4OH^−^ (cathodic reaction) (2)

As predicted in the Pourbaix diagram, ferrous hydroxide is the most common corrosion product due to the reaction:

Fe^2+^ + 2OH^−^ → Fe(OH)_2_ (products of the reaction) (3)

Other common degradation products of Fe corrosion at physiological pH are the coordination compounds of iron oxides (FeO ∙ Fe_2_O_3_). Since the human body environment is full of sodium, chloride, carbon and phosphorus, other products of iron degradation, like iron carbonates, iron phosphates, and iron chlorides, were observed [34]. That’s why Qui et al. modified the Pourbaix diagram for Fe in an aqueous environment to consider the environmental conditions that the implant encounters in the body (T = 37 °C., [Fe^2+^] = 1 × 10^−6^ mol/dm^3^, [Fe^3+^] = 3 × 10^−5^ mol/dm^3^, *p* (O_2_), = 13.3 kPa [35]. This version of the diagram can be very useful when planning iron degradation experiments in a physiological environment.

Kraus et al. presented studies on the corrosion of pure iron after a 52-week in vivo test [36]. It was found that the corrosion product initially consisted of a single oxide layer but became thicker over time to form two layers of a corrosion film. The first layer adjacent to the Fe surface consisted of iron and oxygen. The second layer would form initially after the oxidation of the reaction product from Equation (3) to form stable Fe(OH)_3_:

Fe(OH)_2_ + 1/2H_2_O + 1/4O_2_ → Fe(OH)_3_ (4)

It is worth noting here that very often, the simulated corrosion time based on in vitro tests does not coincide with the results from in vivo tests. This is due to the fact that, under the conditions of the pseudo-physiological environment, not all factors influencing the course of corrosion are taken into account:

(1) Proteins: All pseudo-physiological fluids are designed to simulate the pH and ionic concentrations of blood plasma. However, they do not contain proteins, which are the most important component of blood plasma. Proteins can bind metal ions and then transport them from the implant surface, but also they could create a layer of adsorbed proteins, which then works as a barrier between the metal surface of the stent and the environment, thus inhibiting biodegradation. This same layer in slightly different conditions could also limit the diffusion of oxygen to certain regions of the surface and, in this way, cause a breakdown of the passive layer and preferential corrosion of oxygen-deficient regions [37].
(2) Atmosphere: most degradation tests are carried out in air. However, in blood vessels, there is an atmosphere containing around 5% of CO_2_, whose influence could change the type of degradation products [38].
(3) Cells: During healing of the wound caused by the implantation operation, the surface of the implant is covered with a layer of cells after a few weeks. When the barrier formation is complete, the implant functions in a different environment than before. This may be the main reason for the differences in long-term in vivo and in vitro corrosion [25].
(4) Dissolved oxygen: Blood has about 3 cm^3^/dm^3^ of dissolved oxygen, (4.3 mg/dm^3^) while, for example, most solutions used in cell-based tests (Hank’s solution) have almost twice the level of dissolved oxygen—about 8 mg/dm^3^. This factor is probably the most important of all, and nevertheless it is very often omitted when simulating corrosion [35].

### Corrosion Tests In Vitro

To fully understand the corrosive properties of an iron-based material for applications in a physiological environment, two different types of in vitro testing can be performed: immersion testing and electrochemical testing.

Immersion tests are divided into static and dynamic types. In a static immersion test, regulated by the ASTM G31 standard, the sample is suspended in a suitable pseudo-physiological fluid (mimicking the ionic composition of the physiological environment to which the test material is to be targeted) for a specified period, usually between 1 and 4 weeks [39]. The corrosion obtained from this test is measured in terms of mass loss. This mass loss is related to a time-related unit through the following equation:

DR = kW⁄Atρ(5)

where: W = mass loss [g]; A = specimen surface [cm^2^]; t = exposure time [h]; k = conversion constant for computing the degradation rate whose actual value depends on the units of the variables. Usually, DR is measured in mm/year—in this situation k = 87,600.

Unfortunately, the static immersion test doesn’t consider the influence of the blood flow inside the artery on the stent, which is really important when cardiovascular devices are tested. This is the reason to use a dynamic immersion test by using (mainly polymeric) tubing for circulation, and a volume for the tested device, where the flow can be controlled in order to simulate the shear stress that the stent is subjected to under real conditions.

The most common system for electrochemical tests uses potentiodynamic polarisation (PDP), which enables the determination of the polarisation resistance of an iron-based material by performing a scan in a small potential range. The obtained data make it possible to calculate the current density, and then, by using this information under the ASTM G59 standard, the rate of material degradation can be estimated [40]. Alternatively, electrochemical impedance spectroscopy (EIS), according to ASTM G106, can also be used to model material degradation using equivalent electrical circuits [41].

## 3. Iron Metabolism

Iron is indispensable for life because it plays a crucial role in a wide range of vital biochemical activities. These activities include oxygen sensing, transport, short-term oxygen storage, catalysis, electron transfer and energy generation [42]. These functions are based on the chemical properties of iron, which can form a variety of coordination complexes, including those with organic ligands. These complexes are both dynamic and flexible. The redox potential between ferrous Fe(II) and ferric Fe(III) cations corresponds to the energies required to drive many biological reactions. The bioavailability of iron is limited because soluble Fe(II) (heme) is readily oxidised to Fe(III) (non-heme iron) under aerobic conditions, and Fe(III) is virtually insoluble under physiological pH conditions. The ability of Fe(II) to donate electrons and Fe(III) to accept electrons in the cellular environment is not only the basis of many biochemical reactions needed by the body but also poses a biological hazard [42]. Iron can be the initiator of reactions in which products are injurious radicals [43]. Catalytic quantities of iron produce hydroxyl radicals (OH^−^) from superoxide (O_2_^−^) and hydrogen peroxide (H_2_O_2_). Redox-active iron also catalyses the generation of reactive organic species, including alkoxyl (ROS), thiyl (RS), thiyl-peroxyl (RSOOS) and peroxyl (ROOS) radicals [43]. Free radicals are highly reactive species that can promote oxidation, and subsequent modification, of proteins and nucleic acids. An increase in the steady state levels of reactive oxygen species, termed oxidative stress, can lead to a variety of inflammatory, neurodegenerative, or ischemic processes. It can also accelerate ageing of the body by accelerating tissue degeneration [43]. A deficiency of iron can lead to organism disorders and diseases [44,45]. In young children, iron deficiency causes disturbances in neurological and psychosomatically development, reduces learning efficiency, and may increase the likelihood of autism [46]. In adults, it causes fatigue, increases the risk of depression and impairs the thyroid gland. Iron deficiency is associated with adverse pregnancy outcomes, like increased maternal illness, low birth weight, prematurity, and intrauterine growth restriction [47].

As iron metabolism is similar in all mammals, animal studies give reliable results and predict long term implant behaviour in the human body [42]. The total body weight of a normal adult person contains 3–5 g of iron [42]. Most of this iron is assigned to erythroid precursors for the production of haemoglobin and mature erythrocytes [48]. The haemoglobin found in each erythrocyte accounts for over 1 billion atoms of iron. The total erythroid contains 30 mg of iron/kg body weight. The liver stores most of the rest of the body’s iron. Iron enters the body with food by absorption by enterocytes in the duodenum. Enterocytes contain ferric reductase, which is an enzyme that ensures that iron is available in its ferrous state. The low pH in the gastric efflux facilitates absorption, which then reduces Fe(III) to Fe(II) and delivers it to a proton-rich milieu where it can be sent for further processing or storage [42].

Most of the absorbed iron will reach the plasma [48], while the protein ferritin provides safe storage of the iron fraction retained by the cell. When the lifespan of the erythrocyte ends, it is shed through the gastrointestinal tract together with any remaining stored iron. This represents a significant mode of iron release and loss from the body. Other mechanisms of iron release involve sweating, bleeding and excretion through the epidermis [42]. These alternative mechanisms are important as there are no regulated processes for iron excretion through the liver or kidney in humans. Therefore, there are limited excretion pathways for the iron ions released from a bioabsorbable implant. However, when the body’s optimal iron levels are exceeded, the body can self-regulate, e.g., by lowering iron intake from food [48]. This brief overview of human iron uptake and excretion leads to the question: Which pathway(s) will be taken by the iron originating from implants to exit the body?

## 4. Biological Properties of Iron-Based Materials

Biocompatibility is the property of a material to fulfil its function in the host organism by interacting with living systems without any mechanical injury, toxicity to adjacent or distant tissues, or rejection by the immune system [49,50]. In the case of biodegradable materials, there is a need to consider the biocompatibility not only of iron implants but also their corrosion products. This biodegradability should be considered at several levels.

### 4.1. Cellular Biocompatibility 

Biocompatibility on the cellular level requires that the presence of the biomaterial is not toxic for building the artery wall with endothelial cells and cells in the blood [49]. For this reason, endothelial cells, blood cells, or smooth muscle cells (SMC) are used as the basis for cytotoxicity stent material studies [25].

For example, Huang et al., in an in vitro cytotoxicity study on silver implanted pure iron, used murine fibroblast cells (L-929), human vascular smooth muscle cells (VSMC) and human umbilical vein endothelial cells (EA. hy-926) [51]. Chang et al. used the same set of cell lines in their research [52]. Also, Čapek et al. used the murine fibroblast L929 cell line in their study [53]. In an analogical test, Liu et al. used human umbilical vein endothelial cells (ECV304) and rodent vascular smooth muscle cells [54]. Another method was chosen by Paim et al. using extracted adipose-derived stem cells from the abdominal adipose tissue of a healthy adult donor undergoing tumescent liposuction [55]. The cells were next subjected to washing and centrifuge procedures.

Cytotoxicity tests can be introduced both: in direct contact of the implant with cells, as well as in indirect contact. Since the cell colony environment usually has a small, closed system with a pseudo-physiological fluid, this may affect the corrosion of the implant by the high concentration of corrosion products. There is a constant washout into the bloodstream, which is why this problem does not occur in in vivo tests. Therefore, usually, in vitro research is carried out without direct contact [56].

The IC_50_ index is widely utilised to indicate. Here, the IC_50_ is the concentration of metal required to kill 50% of cells, which gives a measure of the toxicity of the element at the cellular level [57]. Using a 50 µg/mL concentration of Fe, Zhu et al. observed no cytotoxic effect after one day on human endothelial cells [33]. Assuming that the stent completely degrades in an adult, 50 kg human with a circulating blood volume of about 2800 mL, the blood iron concentration will not exceed 7 µg/mL, hence no short-term risk of cytotoxicity. The more stringent standard is ISO 10993-5, which sets the cytotoxicity limit at the level of 30%. If the cell viability is reduced by more than 30%, the material is considered cytotoxic. Based on this recommendation, Paim et al. proved that iron is not cytotoxic and can be used as a material in biodegradable stents [55].

### 4.2. Tissue Biocompatibility

When the stent is implanted into the body during a surgical operation, a cascade of the following successive responses takes place: at first—injury, then—blood-material interactions, provisional matrix formation, acute and chronic inflammation, granulation tissue development, foreign-body reaction (FBR) and fibrosis. It is also accompanied by thrombus formation involving activation of the extrinsic and intrinsic coagulation systems, the complement, the fibrinolytic, and the kinin-generating systems, and platelets. A temporary “scaffold” is created around the implant with a sustained release of bioactive substances from the stent to accelerate the various stages of wound healing. This location is followed by short-term acute inflammation that then becomes chronic. Chronic inflammation around the implant usually lasts from one to two weeks. If it lasts longer than three weeks, it usually means infection [58]. It is essential to know these basics when performing in vivo testing to see when the body’s response is unnatural and needs to be recorded. At this point, nobody reported problems with infections or rejection of the implant due to the presence of iron [55]. There is no indication of problems with the compatibility of tissues with iron. Simultaneously, during long-term in vivo tests in animals, several teams reported that the degradation of iron causes staining of surrounding tissues [31,37,59]. Degradation products may build up in the vessel walls. However, the coloured tissues did not show any infectious responses or other damage. Long-term animal studies have shown that tissue colour returns to normal after 53 months. Liu et al. suggested that macrophages clean the corrosion product before entering the lymphatic system and finally travelling to the lymph nodes [60].

### 4.3. Hemocompatibility

Since stents are used in the cardiovascular system, it is crucial to determine whether they have a negative effect on the blood. That is why platelet adhesion and hemolysis are investigated. Platelet adhesion is regulated by the ASTM F756-13 standard [61]. It determines how many platelets adhere to the material. If the number of adherent platelets is too high, the implant may be thrombogenic and may cause the formation of blood clots. The hemolysis test checks that the implant does not accelerate blood hemolysis by more than 5% with reference to the negative test. 5% is the value specified by the standard [61]. Usually, for the hemocompatibility tests, either human blood is taken from volunteers or animal blood, e.g., rabbit or sheep blood, is used [51,62,63,64,65,66].

Iron is hemocompatible since few platelets adhere to it, and the hemolysis rate is well below 5% [55]. The problem with increased platelet adhesion may arise if Zn or Ag are added to the alloy [51,67]. This fact is fascinating, especially because pure zinc does not adhere to platelets, and silver is added to many other biocompatible materials that does not affect their thrombogenic properties. Perhaps different types of processing, or an investigation of the percentage compositions, would help avoid this effect.

### 4.4. Clinical Biocompatibility

We find that in vivo studies of iron-based materials are performed much less frequently than in vitro ones. This is due to ethical regulations, the high cost of in vivo research, the need for additional equipment and skills, and their long-term nature. Additionally, not all of the developed tools and materials have the potential to provide clear-cut data from in vivo testing. Considering the process of metabolising iron in the body described earlier and the fact that the U.S. Food and Drug Administration does not include iron in its regulations regarding the doses of elements in drugs and medical products due to its low inherent toxicity, we can assume that the presence of Fe does not adversely affect the organs and functioning of the human body [60].

The research carried out so far proves that iron does not affect the host in any way, does not cause enlargement of glands or accumulation of metal in organs [31]. However, more detailed animal studies are needed, especially lasting a few years, in order to understand the long-term effects of iron and its corrosion products in the body. Table 2 summarizes and compares the current results of in vivo tests, taking into account the duration of the tests and the animal models for which they were conducted.

## 5. Production of Iron Biodegradable Devices

Various methods of producing materials for biodegradable devices allow not only different shapes and formats but can also significantly affect the mechanical properties and corrosion rate of the finished implants.

Metals are melted under a vacuum with the resulting product then subjected to various forming and thermomechanical processes in order to achieve the final device. The vacuum applied during the melting procedure minimises inclusion formation and removes occluded or dissolved gases from the material bulk. Casting of the medical device allows a precise shape, and it is a well-established manufacturing procedure [74,75]. Many different techniques are used to obtain new kinds of iron-based biodegradable materials, but only a few have been chosen and described in this article. After a brief description of each of the methods of obtaining biodegradable iron-based systems, the data on the corrosion rate of the described systems are presented in a tabular form.

### 5.1. Spark Plasma Sintering (SPS)

The SPS method is a method of rapid sintering of powdered materials. Unlike other sintering methods, in which the powder is heated using an alternating current, SPS heating the consolidated powder uses periodically repeated direct current pulses lasting from a few to several hundred milliseconds, with low voltage but high intensity (from several to tens of thousands of amperes). SPS process is characterised by a high rate of efficiency, short duration and a lower temperature regime compared to traditional sintering. With the use of the SPS method, it is possible to consolidate metal powders, ceramics, composites, or intermetallic compounds [76]. Spark plasma sintering was used, for example, in the research of Huang et al., to prepare two composite specimens of Fe–5% Pd and Fe–5% Pt [77]. The sintering of the pure starting powders was carried out under vacuum at 1000 °C for a holding time of 5 min. Also, other studies used SPS to produce iron composites with palladium [53]. They sintered powders for 10 min at a temperature of 1000 °C. Cheng and Zheng obtained iron-based materials with W as the second phases [52]. The sintering process was carried out at 950 °C, 40 MPa pressure for 5 min. In Table 3 the data of corrosion rates for Fe-based materials obtained by SPS is presented.

### 5.2. Vacuum Induction Melting (VIM)

The VIM process uses modern induction furnaces to melt metals. The high power installed in the device allows the full charge of the crucible to be melted quickly, minimizing the exposure of the liquid metal to oxygen from the air. Thus, it provides better control over the chemical composition of the final product. VIM is relatively flexible, featuring the independent control of time, temperature, pressure, and mass transport through melt stirring. Its advantages include (1) flexibility due to small batch sizes (2) removal of dissolved gases, for example, hydrogen and nitrogen (3) precise temperature control and (4) low losses of alloying elements by oxidation [79].

The VIM method was used many times. For example, to prepare six binary iron alloy (Fe-X, where X-Mn, Co, Al, W, B, C) ingots for use as coronary stents. The pure elements were mixed in the ratio 97:3 at.%, melted and cast under an argon atmosphere in a VIM furnace [78]. As for the corrosion rate of the systems obtained by the above method, it is comparable to the corrosion rate of the systems obtained by the STS method (Table 4).

### 5.3. Vacuum Arc Melting (VAM)

Vacuum arc melting (VAM) is a process for melting metals developed in 1839. VAM offer quick melting of the cathodic electrode under the high temperature of the direct-current arc. Then the liquid metal is rapidly solidified into an ingot in the water-cooled mold. Thanks to the dual action of vacuum and an electromagnetic field, the inclusion and gas content of the molten metal can be effectively reduced. Therefore, while VIM establishes close control of alloy formation and chemistry, VAM exhibits the desired microstructure due to greater control of the solidification rate [81]. That’s why this method was used to make three alloy ingots of Fe-based bulk metallic glasses (BMG) prepared by melting the appropriate atomic percentages of Fe, Co, Cr, Mo, C, B, Mn and Y [82]. The homogenisation of the sample compositions was performed by remelting each ingot at least five times. The obtained materials were biologically compatible and showed high resistance to corrosion, as it is presented in Table 5. Therefore, their use as biodegradable material is not possible.

### 5.4. Electroforming

Electroforming is a different form of electroplating. In electroplating, metal is dissolved electrolytically at an anode. The resulting metal ions are transported by an electrolyte solution to be placed at a cathode. The difference between electroforming and electroplating lies in the purpose of use for the deposited metal. Electroplating is concerned with taking an existing article and applying a metallic coating. However, electroforming is obtaining metal object by utilising the electroplating process to deposit a metal on or against a master or mandrel.

Electroforming can produce shapes to tight tolerances, good surface finishes and excellent metallurgical properties because the structure of the metallic parts is formed atom by atom and layer by layer. In their research, Moravej et al. found that pure iron fabricated by electroforming technology possessed a high yield strength (YS) (360 MPa) and ultimate tensile strength (423 MPa) [84]. The ductility could be further improved 18% by annealing. Both static and dynamic in vitro degradation tests indicated a faster corrosion rate of electroformed pure iron than that of pure iron produced by casting (Table 6).

### 5.5. Powder Metallurgy (PM)

In short, PM consists of four basic steps: (1) powder preparation, (2) mixing and blending, (3) compacting and (4) sintering. Sometimes, this process accomplished with some secondary operations like coining, impregnation, hot forging, etc. The advantages of PM include ease of carrying out the process, high purity of the obtained products and the fact that metals and non-metals can be mixed in any proportions. That’s why this method was used many times to produce iron-based biodegradable materials [53,55,85]. PM materials usually show higher degradation rates than casted or wrought ones because PM materials contain some porosity that enhances the exposed area of the sample to the corrosion medium, and obtained products may contain oxides whose can change the corrosion rate [53]. For example, Hermawan et al. used PM to prepare four Fe–Mn alloys, namely Fe20Mn, Fe25Mn, Fe30Mn, and Fe35Mn, from high purity elemental powder [85]. The numbers specified the nominal weight percentage of manganese. The sintering temperature of the compacted powder mixtures was fixed at 1200 °C for 3 h in a flowing Ar-5% H_2_ gas mixture. The addition of manganese powder significantly modified the corrosion properties of the systems, increasing their the corrosion rate (Table 7). It should be noted, however, that the optimal manganese addition was determined—and this at the level of 20% turned out to be the most advantageous from the point of view of the planned biodegradability. Higher percentages of manganese powder resulted in a decrease in the corrosion rate of Fe-Mn systems.

### 5.6. Mechanical Treatments, Inducing Plastic Deformation

One of the best methods to obtain pure iron devices with improved mechanical properties is to perform mechanical treatments that induce plastic deformation, such as cold rolling or cold drawing [86]. These methods create a finer grain structure as defined by the Hall–Petch equation. However, these treatments may decrease the ductility of the material [81]. At the same time, they lead to a reduction in the corrosion rate, as compared to unmodified systems (Table 8) [83].

### 5.7. Template-Based Formation of Porous Material

The structure of the material has a great influence on its properties. In the event of a need to accelerate corrosion, one of the most common methods is the creation of a porous material. The mechanical properties of iron allow it to create metallic foams with a high pore per inch (ppi) content while maintaining shape and strength [22]. The most common method of creating porous iron foams is to apply powdered iron with optional metallic additives to a polyurethane (PU) foam base, followed by heating in a furnace at high temperatures until the template is destroyed [87,88].

Porous, iron-based materials degrade faster than their equivalent, fully dense iron counterparts based on size/weight. This is due to the larger contact surface with the physiological environment and more locations where corrosion can be initiated. Porous materials are also more prone to crevice corrosion, which accelerates the material adsorption [89,90]. Studies confirmed the influence of pore sizes on corrosion rate. Heiden et al. noticed that the addition of controlled porosity and hydroxyapatite as the second phase in Fe-30Mn increased the corrosion rate by almost 40 times compared to the non-porous Fe-30Mn (Table 9) [91]. Zhang et al. used as porogen ammonium bicarbonate (NH_4_HCO_3_) in biodegradable Fe-35Mn [92]. The degradation rate was significantly accelerated when compared to samples without porogen. However, the porous, iron-based material did not have satisfactory mechanical properties. Čapek et al. studied a Fe-Pd porosity material prepared by SPS [53]. The porous material showed faster corrosion compared to the solid condition but also scored worse in cytotoxicity tests. This does not preclude it from being used as a biocompatible material but requires additional optimization.

### 5.8. 3D Printing of Iron Materials

Three-dimensional printing has been explored as a fabrication method for patient-specific devices utilizing existing biomaterials. 3D printing of biodegradable metals, such as iron, has the potential to deliver patient-specific biodegradable scaffolds and, depending on the required properties of the scaffold at different implantation sites, a porous structure of the scaffold can be designed and fabricated by 3D printing, for example, to enhance the corrosion rate. The design can also be engineered to specifically load any drugs or biologically active substances.

Currently, electron beam melting and selective laser sintering is widely used for the fabrication of metal parts. Although, the major limitations, such as the use of an expensive 3D printer and availability of materials, may restrict its further application for metal printing [93]. In earlier studies, it was found that micro-extrusion-based 3D printing covered a broad range of available materials and was flexible enough to fabricate metal 3D parts at a low cost [93,94]. Micro-extrusion-based 3D printing is an ink-based dispensing system with a robotic arm that extrudes a continuous stream of ink through a micro-size nozzle [95]. However, Taylor et al. investigated the approach to fabricate porous iron by extrusion-based 3D printing and conventional sintering [96]. The PLGA-based copolymer was used as the binder for preparing the metallic-based polymer ink. After heating the green body in a conventional sintering furnace, a porous metallic structure was formed, although conventional sintering required additional processing time and energy to heat the sample [97]. A six-step approach to fabricate the porous iron by the amalgamation of 3D printing and pressureless microwave sintering has been developed by Sharma and Pandey [98]. This six-step process enclosed the design of a porous structure template by 3D printing, which was further used to fabricate a porous Fe scaffold using microwave sintering. Microwave sintering requires a lower energy consumption to sinter the metal part in less processing time and also results in a better mechanical properties in the sintered part when compared to the conventional sintering process [97].

## 6. Iron Materials Modifications

### 6.1. Additions

One of the most common procedures to increase the degradation rate and modify the mechanical properties of pure iron is through alloying. An additional element can be soluble in iron or dispersed as metal matrix composites (MMC). The electrochemical properties of the additive have the highest influence on the device degradation rate. There are two main ways to reduce the corrosion resistance of iron alloys: (1) the addition of elements with lower electrochemical potential than Fe (−0.44 V versus SHE) and soluble in iron to decrease the corrosion resistance of the matrix; (2) addition of elements nobler than iron to create a second phase with higher electrochemical potential and cause galvanic corrosion, where iron is the anode, and the more noble metal is the cathode [99].

Liu and Zheng tested the influence of the addition of low contents of common alloying elements (Mn, Co, Al, W, Sn, B, C and S) on the corrosion rate and biocompatibility of pure iron [78]. They found that adding Co, W, C, or S slightly accelerated the corrosion of pure iron. Mn, Al, and B slowed the corrosion insignificantly. The results also showed that the addition of all alloying elements, except for Sn, improved the mechanical properties of iron after rolling. It should be emphasised that this study applies to small percentages of the additive in the alloy; larger amounts may give different results. In the afore-mentioned study, there was no influence found for the added elements on the cytotoxicity of the alloy. Cheng and Zheng also conducted research on adding tungsten to pure iron [62]. They confirmed the acceleration of corrosion and no visible effect on the adhesion of platelets under the influence of W added by SPS. In the same study, they suggested the use of a non-metal carbon nanotubes (CNT) as an alloy additive, which increased the corrosion rate and improved the strength.

Due to its higher nobility (+0.951 V vs. SHE), the addition of palladium is fairly often used in research on increasing the biodegradability of iron. Pd can also stabilise iron in the austenite form [100]. The important fact is that palladium is completely miscible in iron. After solubilisation, it improves the mechanical properties of the iron matrix. However, Pd can also be precipitated as the second phase (following the associated phase diagram) or produced as MMC from metal powders under controlled temperatures [101]. For example, Čapek et al. showed that alloying iron with palladium led to an increase in the mechanical properties and corrosion rates [53]. Their porous implant, with a diameter of 5 mm obtained by SPS, should fully degrade after approximately 5 years. Gao et al. used palladium as the second phase in the iron matrix with selective laser melting used to prepare the samples [102]. The obtained biocomposite degraded faster than pure iron through microgalvanic corrosion between the noble Pd-rich intermetallic phase and the Fe matrix.

Platinum exhibits many properties similar to palladium since they belong to the same group in the periodic table, but the electrochemical potential of Pt is higher than Pd (+1.18V vs. SHE). Huang et al. compared Fe-5wt.%Pd and Fe-5wt.%Pt composites [77]. The addition of either elements sped up the corrosion process of iron, and improved the mechanical properties compared to pure iron. However, better results were given by modification with platinum Pt added as the second phase to the iron as it improved the yield strength, rigidity and microhardness nearly three times that of pure iron [77].

Gold is one of the noblest metals (+1.69 V vs. SHE) and has a long history used as a dental material [101]. Hulander et al. shown that the ground state cytotoxic activity of Au is minimal and can be ignored, but Au salts are toxic to cells [103] Gold is partially soluble in iron, so this makes the second phase possible but under controlled conditions [104]. Huang et al. fabricated Fe-Au composites by SPS with a variable percentage of gold [77]. They noticed that the iron-based composites with 5 wt% of Au (or Ag) became the materials with the fastest corrosion rate after 30 days of immersion. All composites degraded much faster than pure iron. At the same time, no increase in cytotoxicity or platelet adhesion was found.

Silver is a popular choice as an addition to manufacturing traditional medical devices due to its good biocompatibility and antibacterial properties [105,106]. Ag has a higher electrochemical potential (+0.800 vs. SHE) than iron, thanks to which it can cause galvanic corrosion. In contrast to gold, silver is not soluble in iron [104]. Huang et al. showed that the addition of Ag to pure iron resulted in faster corrosion than the addition of Au as the second phase [107]. In these tests, silver did not show any cytotoxicity or thrombosis effect. Loffredo et al. tested the difference between Fe-16Mn-0.7C alloy and Fe-16Mn-0.7C-0.4Ag [108]. As a result, it was shown that the presence of silver decreased the ductility caused by the development of mechanical ε-martensite. More about martensite is found below in the Section 6.2 “Iron-Manganese alloys”. In contrast to other results in the study, Čapek et al., who added 2 wt% silver to pure iron, found that Ag deteriorated the corrosion rate of Fe in both [109]: (1) the long-term immersion test and (2) potentiodynamic test.

Wang et al. noted that the addition of Ga appears to significantly improve the degradation rate of pure Fe by 5 times [110]. The primary mode of corrosion in the alloy was pitting corrosion. They suggested that, due to the more negative electrochemical potential of Ga (−0.56 V vs. SHE) compared to Fe, a more negative corrosion potential was obtained in the alloy. Also, the heterogeneous structure of the alloy would have been conducive to the occurrence of microgalvanic corrosion.

Cheng et al. researched the influence of the addition Fe_2_O_3_ (in variable contents) to pure iron obtained by SPS [52]. As a result, they found no significant difference in the corrosion process, cytotoxicity or platelet adhesion between composites of Fe_2_O_3_-Fe and pure iron.

Promising results were achieved by adding nitrogen to iron [111]. W. Lin et al. published a series of studies about nitriding iron stents [70,112,113]. Nitrided iron turns out to corrode faster than pure iron and has better mechanical properties. Long-term in vivo studies have shown the alloys to be fully biocompatible.

### 6.2. Iron-Manganese Alloys

As mentioned before, Mn is often an addition to iron biodegradable materials to improve the mechanical properties and biodegradability. This was started in 2007 by Hermewan et al. through the publication of research on the iron-35 wt% manganese (Fe-35Mn) alloy and suggested this alloy as a promising option due to its low magnetic susceptibility and suitable corrosion behaviour [114].

Manganese is known as an important element in the human body. Its highest concentration is in the bones, liver, kidney, pancreas and adrenal gland. In normal, healthy humans, blood Mn concentrations range from 4 to 15 μg/L, which means that the human body has a good reaction to the presence of manganese in implants. Notwithstanding, a long exposure to too high a dose of Mn is highly toxic to the body [115]. Manganese has a function as a neurotransmitter in the brain, that’s why intoxication by this element induces the first signs and symptoms in the nervous system. There is evidence that long-term exposure to manganese could induce Parkinson’s disease [116,117]. This intoxication has also a significant effect on cardiac function: it inhibits myocardial contraction, dilates blood vessels, and induces hypotension [115]. Therefore, in the design of Fe-Mn biodegradable cardiovascular devices, particular attention should be taken to prevent the local accumulation of manganese ions during material corrosion. Moreover, deficiency of iron ions increases the likelihood of absorption of excessive amounts of manganese and thus its intoxication, which suggests that the presence of iron in Fe-Mn alloy could prevent Mn corrosion toxicity [115,118]. On the other hand, manganese deficiency, even if rare, disrupts the healthy functioning of the central nervous system and reproductive system, as well as impedes growth.

Manganese physical properties are similar to iron. Its electrochemical potential is lower than iron (−1.18 V vs. SHE) and both these elements are soluble in each other [104]. That’s why the Fe-Mn alloy has a more active corrosion potential than pure Fe.

The degradation process of manganese is similar to that described before for iron degradation [80]:

Mn → Mn_2_^+^ + 2e^−^ (anodic reaction) (6)

O_2_ + 2H_2_O + 4e^−^ → 4OH^−^ (cathodic reaction) (7)

Mn^2+^ + 2OH^−^ → Mn(OH)_2_ (product reaction) (8)

However, the human artery atmosphere has around 5% more CO_2_ than in the ambient atmosphere. This is an important factor in Mn degradation because Mouzou et al. showed additional reactions disturbing the corrosion process of the alloy in an atmosphere rich in CO_2_ [38]:

CO_2_ + H_2_O → H_2_CO_3_ (9)

2H_2_CO_3_ → 2H^+^ + 2HCO_3_^−^ (10)

2HCO_3_^−^ → 2CO_3_^2−^ + 2H^+^ (11)

Mn → Mn^2+^ + 2e^−^ (12)

Mn^2+^ + 2HCO_3_ → Mn(HCO_3_)_2_ → MnCO_3_ + CO_2_ + H_2_O (13)

Mn^2+^ + CO_3_^2−^ → MnCO_3_ (14)

The same type of reaction also describes oxidation of iron present in the alloy:

Fe^2+^ + 2 HCO_3_ → Fe(HCO_3_)_2_ → FeCO_3_ + CO_2_ + H_2_O (15)

Fe^2+^ + CO_3_^2−^ → FeCO_3_ (16)

Due to the fact that FeCO_3_ and MnCO_3_ have a low solubility in an aqueous environment, and they can form a stable passivation layer at the surface of the device, carbonates can block the degradation process [38].

Another interesting feature of manganese is that it stabilises the austenitic allotrope in a large range of compositions, which helps to avoid ferromagnetism in devices. If in a composition that is more than 15% Mn then antiferromagnetic behaviour will be observed in all alloys, since, as the second phase occurs as ε-martensite. However, a contribution can still be observed in the presence of α-martensite, which shows a ferromagnetic behaviour [119,120,121]. The accelerated degradation of Fe-Mn alloys with different Mn content in relation to pure iron has been confirmed in many studies [38,69,89,114,122,123,124,125].

To improve the Fe-Mn combination properties, a third component can be introduced to produce a Fe-Mn-X alloy. The literature gives many different examples of these modifications. Generally, there are several basic types of alloy additives:

(1) Noble metals as a second phase composite, like Pd [36,126,127], Cu [73,128], Ag [125,129,130] cause local galvanic corrosion, because of their high electrochemical potential.
(2) The addition of silicon drew the attention of scientists because devices made of the Fe-Mn-Si alloy have shape memory and a higher corrosion rate [54,68,72,83,131,132].
(3) Elements from the second group, Mg and Ca, have strong negative electrochemical potentials (Mg = −2.38 V vs. SHE, Ca = −2.76 V vs. SHE). In addition to a Fe-Mn matrix creates alloys possessing active corrosion potentials. After analysing Tafel curves, Hong et al. suggested that the Fe-Mn-Ca alloys have a greater tendency to corrode than the Fe-Mn-Mg alloys [133].
(4) The addition of carbon to Fe-Mn has an alloy composition that aligns with that of the twinning-induced plasticity (TWIP) steels. TWIP steels have excellent strength and ductility. The studies confirmed that TWIP (Fe-Mn-C) alloys degrade in a physiological environment [134,135,136,137].

Venezuela and Dargush lately wrote a more detailed review only about Fe-Mn biodegradable alloys, to which I refer to those interested [138].

### 6.3. Surface Modification

Concerning the number of studies carried out to change the composition or the method of obtaining an iron-based biodegradable device, the number of studies on surface modifications is small. However, the existing results indicate that this may be the most appropriate method for implants with improved performances. Zhu et al. covered pure iron with a thin film of Fe-O by plasma immersion ion implantation and deposition to improve the mechanical integrity and help with biocompatibility [139]. As a result, the prepared films reduced the number of adhered platelets and restrained the aggregation but also improved the surface corrosion resistance. Sandblasting the iron surface was tested by Zhou et al. which changed in an early state the surface composition, increased the density of dislocations and roughness [140]. They all investigated biocompatibility and corrosion rate. Huang and Zheng applied photolithography and electron beam evaporation of platinum discs on an iron surface, and in this way, they decreased the corrosion potential [141]. Chen et al. in their research, suggested a calcium zinc phosphate coating to delay the start of iron corrosion in the first stage, during wound healing, and to improve biocompatibility [142].

Huang et al. reported that Zn ions, implanted by a metal vapour vacuum arc in Fe, accelerated the degradation rate of the iron [67]. Also, Wang et al. used the ion implantation technique to inject Zn onto the surface of biodegradable, pure Fe [143]. In both examples, corrosion resistance decreased in comparison with pure iron and caused pitting corrosion. However, additional zinc on the surface of implantation devices enhanced the adhesion of platelets compared to pure Fe in both static and dynamic immersion tests in Hank’s solution [67].

Cheng et al. created Au patterns on pure Fe using vacuum sputtering to deposit Au on the substrate [63]. They observed that the Fe with Au exhibited a higher corrosion rate than pure Fe. Furthermore, the Au pattern seemed to induce more uniform corrosion of the substrate. As expected, the corrosion film loosened the gold’s adhesion to the surface and caused the disk to be eventually displaced.

Weng et al. attempted to modify the iron surface by tantalum ion implantation [144]. The results showed that Ta/Fe oxide mixtures were formed on the outermost surface of the modified layer after ion implantation. After modification by the Ta ion, pure iron exhibited a higher corrosion rate due to the formation of severe pitting corrosion. During tests, cells showed an enhanced adhesion and proliferation behaviour on the surfaces of Ta implanted Fe.

In other studies, Huang et al. showed that silver ions were implanted into a pure iron surface by metal vapour vacuum arc technique [51]. The Ag layer was about 60 and the highest content of Ag was 5 at.%. On the outer implantation layer, Ag existed as Ag_2_O. With the increase of depth, Ag gradually changed from Ag_2_O to Ag elementary substance. In comparison with pure iron, the surface modified by silver exhibited faster and much more uniform corrosion. However, Ag ions slightly decreased the viabilities of experimental cells and increased the risk of thrombosis.

### 6.4. Coatings

Promising results were given by coating iron devices with bioresorbable polymers because during degradation, they locally acidify the environment around the implant. The study of Yusop et al. demonstrated that iron corrosion could be expedited by the hydrolysis of PLGA (poly(lactic-co-glycolic acid)) that produced soluble monomers consisting of carboxylic acid groups, which could lead to a pH decrease [145]. Qui et al. studied the behaviour of an iron stent covered in polylactic acid (PLA), and a strut with a thickness of 50 µm was completely dissolved after 6 months [18]. Studies by Lin et al. also showed promising results with the use of PLA [113]. Recently, research by Gorejová et al. about coated iron foams with three different concentrations of PEI (polyethyleneimine) showed that the coverage on the iron surface by PEI gives a desirable corrosion rate enhancement mediated through polymer cracking and corrosion medium penetration [146].

In an investigation by Huang et al., they checked the corrosion behaviour of collagen-coated porous and nonporous Fe-Mn [147]. In both porous and non-porous alloys, the collagen coating reduced the corrosion rate as the protein prevented direct contact of oxygen with the iron surface.

Drevet et al. studied the influence of pulsed electrodeposited calcium phosphate coatings on FeMnSi alloys with variable percentage composition [148]. In every example, the corrosion rate of the alloy was reduced. A similar effect was achieved by applying an osteoconductive hydroxyapatite-zirconia coating on FeMnSi by using pulsed laser deposition in research carried out by Cimpoesu et al.—the ceramic coating protected the substrate with a decreased corrosion rate for the alloy [149].

Hydroxyapatite has been used several times for iron coating; however this material appears more often in works on bone implants due to it being known to be biocompatible with bone tissue [34,150].

### 6.5. Drug Delivery from Iron-Based Stents

A major problem with bare metal stents is restenosis due to neointimal overgrowth. To resolve this problem, drug eluting stents with immunosuppressive or cytotoxic drugs were designed to inhibit neointimal hyperplasia. Usually, drug loading is from a polymeric coating this makes it easier to control the elution kinetics. Controlled and sustained release of the drug in the first 10–30 days after transplantation, during healing wound after surgery, is critical to the device’s anti-restenotic effectiveness [151]. In the case of iron-based BRS, the polymeric coating must also be fully biodegradable. 

The most commonly used drugs in cardiac stents, among others, are (1) Curcumin, as an inhibitor platelet aggregation [152]; (2) sirolimus to inhibit inflammation, proliferation and cell migration [153]; (3) estrogen to promote vascular healing [154]; (4) angiopeptin as a redactor of neointimal hyperplasia [151]; (5) dexamethasone, to reduce inflammation [155]; (6) tranilast to reduce neointima formation and inflammation [155].

In studies, Yusop et al. used poly-L-lactic acid with different curcumin amounts as a coating for iron scaffolds [156]. This research looked at bone implants; hence the role of curcumin is to retard the growth of osteosarcoma cells owing to cancer arrest. It turned out that the iron degradation process may affect the kinetics of curcumin release. From the point of view of mechanics, the CP particle stiffness and interfacial interactions between the CP coating and the Fe surface further enhanced the mechanical strength of CP-Fe. After 28 days, the scaffold showed good strength reduction. 

Shi et al. conducted in vivo studies on rats, checking the biocompatibility and corrosion rate of the sirolimus-eluting iron bioresorbable coronary stent system [157]. After 90 days, the iron was at an early stage of corrosion. 

Cysewska et al. tested the release of anti-inflammatory salicylate from polypyrrole (PPy)-coated iron [158]. Sodium salicylate was incorporated into PPy by one-step electropolymerisation. As a result, depending on different conditions, they obtained materials with different morphologies and redox properties. The drug-release study showed that, during polymer degradation, the release of salicylate proceeds efficiently.

Sharipova et al. took a different approach to the topic because they did not use the polymer as a drug-eluting medium [159]. Instead, the iron powder was mixed with 10% Ag and VH as a model drug (used for the treatment of periprosthetic or implant-related infections). Afterwards, the mixed powder mixtures were cold-sintered in tungsten carbide dies at RT by utilising a single-stage press. In in vitro tests, the tested material killed pathogens associated with chronic bone infections after 5 days. At the same time, studies showed no iron-based material cytotoxicity.

Nitric oxide is among other substances that positively influence the treatment process because it is an important molecule in biological systems and plays a critical role in pathophysiology and disease [160]. This resulted in the development of new therapeutic strategies for nitric oxide. It may mean that nitriding iron stents, in addition to good mechanical and corrosive parameters, can also have a beneficial effect on the adjacent tissue.

## 7. Conclusions and Future Perspectives

The excellent biocompatibility of iron alloys and their high strength, ductility, and—especially—elastic modulus compared to magnesium and zinc alloys make iron one of the best potential materials for creating biodegradable metallic stents since the beginning of the 21st century. Due to its mechanical properties and workability, it could be used to create lighter and smaller stents.

The problem encountered by researchers working with iron is the slow corrosion time in vivo, which prevents the recognition of iron as fully biodegradable. The challenge will be to create new, iron-based materials that, without losing their advantages, will have a higher corrosion rate. For this purpose, research is carried out on alloy additions, the use of various manufacturing techniques to modify the structure and surface morphology of the material or the use of additional materials as a coating to influence the course of corrosion.

The analysis of data on the rate of degradation processes of iron, its alloys, or iron-based systems and a critical comparison of the numerical values of this property allows us to conclude that some of the methods of obtaining potential ferrous biomaterial, such as spark plasma sintering, and vacuum induction melting, as less effective, should be abandoned. And a few others, including vacuum arc melting and electroforming, should be looked at once again in detail to optimize them further. Both powder metallurgy and templat-based porous systems synthesis have the potential to produce almost optimally degrading iron-based systems, and further work to achieve the desired iron-based systems should be planned. Of course, it is also possible to continue the work on introducing an additional phase into the iron and creating new alloys, but then it should be borne in mind that among the degradation products, there will be systems of more than one element, which may lead to a greater probability of negative side effects. In the case of staying with pure iron, it is possible to focus only on optimising the degradation rate related directly to the morphology of the system (its porosity, specific surface), excluding the influence of other elements. However, the correct solution to obtain optimally degraded Fe-based material is still unknown. After reaching described in the review milestones, there is still much work to do. With the development and dissemination of new manufacturing and imaging technologies, it will be possible to produce individual devices that will be perfectly adapted to the needs of a particular patient in terms of their composition and shape. Also, the size and weight of the stents will be possibly decreased without losing their mechanical strength to limit their harmful effects on the blood vessel walls. Another way to go further is to optimise drug release in stents and propose new solutions for personalised therapy.

## Figures and Tables

**Table 1:** The mechanical properties of biodegradable metals.

	Yield Strength (MPa)	Young’s Modulus (GPa]	Tensile Strength (MPa)	Shear Modulus (GPa)	Elastic Modulus (GPa)	Hardness (HV)	Ref
Mg	51	44–45.5	175–235	16–18	44–48	38	[26,27]
Zn	285–325	90–110	90–200	35–45	14–32	42	[28]
Fe	108–122	204–212	230–345	78–84	195–235	157	[29,30]

**Table 2:** Summary of published in vivo test.

Material	Shape	Animal	Implantation Place	Duration	Results	Ref
Pure iron	Stent	White rabbit	Native descending aorta	6, 12, 18 months	Toxicity wasn’t observed, there was no neointimal proliferation and no excess inflammatory reaction	[31]
Pure iron	Zig-zag stent	Domestic swine	Left coronary ostium	28 days	Stents started degradation without signs of thrombosis or immoderate inflammation	[59]
Pure iron	Discs	Rats	Dorsal area	1 week, 3, 6 months	Lower degradation rate of iron samples in vivo than in vitro	[55]
Fe28.5Mn28.5Si	Rectangular	Wistar rats	Bone (tibiae) and subcutaneously (back)	14, 28 days	Concentration of iron at implant surface after 28 days significantly decreased in bone and subcutaneous implants, mass of bone implant decreased after implantation whilst mass of subcutaneous implant increased	[68]
Fe	Pin	Rats	Femoral bone	4, 12, 24, 52 weeks	Fe ions from degradation were transported 1mm into tissue surrounding implant, but no local toxicity	[36]
Fe10Mn1Pd	Pin	Rats	Femoral bone	4, 12, 24, 52 weeks	No significant change to degradation rate of implant, no local toxicity	[36]
Fe21Mn0.7C1Pd	Pin	Rats	Femoral bone	4, 12, 24, 52 weeks	Slower degradation rate than previous samples, but change was not significant, no local toxicity	[36]
Fe30Mn	Wire	Rats	Femoral bone	6 months	Bone on growth was observed for bone in contact with implant, alloy did not cause adverse effects, and an iron oxide layer was observed on the implant surface	[69]
Fe-5%HA composite	Plate	Sheep	Tibiae	3, 9, 14, 35, 50, and 70 days	Degradation rate was slower than degradation rate of pure iron implant	[34]
Fe-5%TCP composite	Plate	Sheep	Tibiae	3, 9, 14, 35, 50, and 70 days	Higher degradation rate in contrast with pure iron implant	[34]
Fe-3%HA-2%TCP composite	Plate	Sheep	Tibiae	3, 9, 14, 35, 50, and 70 days	Increase of radiopacity of implant on day 35, probably caused by accelerated bone growth and healing of tissues, degradation rate higher than pure iron implant	[34]
Fe0,074N	Stent	Rabbit, Minipigs	abdominal aorta, left anterior descending, right coronary artery	12, 36 months	Nitride modified scaffold showed non-uniform corrosion, higher corrosion rate after implantation in contrast with pure iron stent, no unusual reactions, no pathological changes to tissues	[70]
FeXMn (X = 0.5, 2.7, 6.9)	Cylindrical plate	Mice	Back (subcutaneous)	3, 6, 9	There was no significant corrosion after implantation. Implants degraded slowly probably, because of phosphate layers on surface of corroding implant	[71]
Fe35Mn1Ag	Rods	Rats	Subcutaneous	4, 12 weeks	The addition on silver increases corrosion rate in vivo, almost two times higher rate than Fe35Mn alloy	[72]
FeMn10Cu	Rods	Rabbit	Femur	30, 90 days	Addition of copper increased corrosion rate in vivo in comparison to base FeMn alloy	[73]

**Table 3:** Corrosion rates for Fe-based materials obtained by spark plasma sintering.

Method	Material	Corrosion Rate (mm/Year)	Ref.
SPS	Fe	0.016	[52]
	Fe2W	0.075	[52]
	Fe5W	0.138	[52]
	Fe35Mn5Si	0.025	[77]
	Fe25Mn10Cu	0.258	[73]
	Fe34Mn1Cu	0.032	[73]
	Fe	0.105	[78]
	Fe3Mn	0.105	[78]

**Table 4:** Corrosion rates for Fe-based materials obtained by vacuum induction melting.

Method	Material	Corrosion Rate (mm/Year)	Ref.
VIM	Fe3Co	0.128	[78]
	Fe3Al	0.112	[78]
	Fe3W	0.151	[78]
	Fe3B	0.175	[78]
	Fe3C	0.187	[78]
	Fe3S	0.145	[78]
	FeMnC	0.13	[80]
	TWIP1Pd	0.21	[80]
	Fe	0.11	[80]

**Table 5:** Corrosion rates for Fe-based materials obtained by vacuum arc melting.

Method	Material	Corrosion Rat (mm/Year)	Ref.
VAM	Fe	0.027	[51]
	Fe implanted Ag	0.046	[51]
	Fe	0.1	[83]
	Fe30Mn	0.24	[83]
	F330Mn5Si	0.76	[83]
	F3e26Mn5Si	0.56	[83]
	Fe23Mn5Si	0.44	[83]

**Table 6:** Corrosion rates for Fe-based materials obtained by electroforming.

Method	Material	Corrosion Rate (mm/Year)	Ref.
Electroforming	Fe	0.4	[83]
	Fe	0.85	[84]

**Table 7:** Corrosion rates for Fe-based materials obtained by powder metallurgy.

Method	Material	Corrosion Rate (mm/Year)	Ref.
PM	Fe20Mn	1.3	[84]
	Fe25Mn	1.1	[84]
	Fe30Mn	0.7	[84]
	Fe35Mn	0.4	[84]
	Fe	0.2	[84]

**Table 8:** Corrosion rates for Fe-based materials obtained by powder metallurgy followed by cold rolling.

Method	Material	Corrosion Rate (mm/Year)	Ref.
PM cold rolled	Fe20Mn	0.5	[84]
	Fe25Mn	0.5	[84]
	Fe30Mn	0.7	[84]
	Fe35Mn	0,7	[84]

**Table 9:** Corrosion rates for porous Fe-based materials.

Method	Material	Corrosion Rate (mm/Year)	Ref.
Template-based formation of porous material	Fe	1.18	[90]
	Fe30Mn10HA	0.82	[91]

## Data Availability

Not applicable.
